# Long non-coding RNA CASC2 suppresses pulmonary artery smooth muscle cell proliferation and phenotypic switch in hypoxia-induced pulmonary hypertension

**DOI:** 10.1186/s12931-019-1018-x

**Published:** 2019-03-11

**Authors:** Junsong Gong, Zujun Chen, Yu Chen, Huanran Lv, Haisong Lu, Fuxia Yan, Lihuan Li, Weili Zhang, Jia Shi

**Affiliations:** 1 Department of Anesthesiology, State Key Laboratory of Cardiovascular Disease, Fuwai Hospital, National Center for Cardiovascular Diseases, Chinese Academy of Medical Sciences & Peking Union Medical College, No.167 Beilishi Rd., Xicheng District, Beijing, 100037 China; 2 Surgical Intensive Care Unit, State Key Laboratory of Cardiovascular Disease, Fuwai Hospital, National Center for Cardiovascular Diseases, Chinese Academy of Medical Sciences & Peking Union Medical College, Beijing, 100037 China; 3 State Key Laboratory of Cardiovascular Disease, FuWai Hospital, National Center for Cardiovascular Diseases, Chinese Academy of Medical Sciences & Peking Union Medical College, Beijing, 100037 China

**Keywords:** Pulmonary hypertension, Pulmonary artery smooth muscle cells (PASMCs), lncRNA CASC2, Vascular remodeling; phenotypic switch

## Abstract

**Background:**

In this study, we aimed to investigate whether and how lncRNA CASC2 was involved in hypoxia-induced pulmonary hypertension (PH)-related vascular remodeling.

**Methods:**

The expression of lncRNAs or mRNAs was detected by qRT-PCR, and western blot analysis or immunochemistry was employed for detecting the protein expression. Cell number assay and EdU (5-ethynyl-2′-deoxyuridine) staining were performed to assess cell proliferation. Besides, flow cytometry and wound healing assay were employed for assessments of cell apoptosis and cell migration, respectively. Rat model of hypoxic PH was established and the hemodynamic measurements were performed. Hematoxylin and eosin (HE) and Masson′s trichrome staining were carried out for pulmonary artery morphometric analysis.

**Results:**

The expression of lncRNA CASC2 was decreased in hypoxia-induced rat pulmonary arterial tissues and pulmonary artery smooth muscle cells (PASMCs). Up-regulation of lncRNA CASC2 inhibited cell proliferation, migration yet enhanced apoptosis in vitro and in vivo in hypoxia-induced PH. Western blot analysis and immunochemistry showed that up-regulation of lncRNA CASC2 greatly decreased the expression of phenotype switch-related marker α-SMA in hypoxia-induced PH. Furthermore, it was indicated by the pulmonary artery morphometric analysis that lncRNA CASC2 suppressed vascular remodeling of hypoxia-induced rat pulmonary arterial tissues.

**Conclusion:**

LncRNA CASC2 inhibited cell proliferation, migration and phenotypic switch of PASMCs to inhibit the vascular remodeling in hypoxia-induced PH.

**Electronic supplementary material:**

The online version of this article (10.1186/s12931-019-1018-x) contains supplementary material, which is available to authorized users.

## Introduction

Pulmonary hypertension (PH) is divided into pulmonary arterial hypertension (PAH), PH resulting from left heart disease, PH resulting from lung disease or hypoxia, chronic thromboembolic pulmonary hypertension and PH of uncertain multifactorial mechanism [1]. PH is a vascular disease characterized by vasoconstriction, cellular hyperplasia, high pulmonary arterial pressure, right ventricular heart hypertrophy and vascular remodeling [2]. It is estimated that more than 100 million people worldwide are affected by PH [3]. The abnormal proliferation and hypertrophy of pulmonary arterial smooth muscle cells (PASMCs) lead to vascular remodeling, which is a central process in the PH pathogenesis [4]. PASMCs are extremely plastic,which can dedifferentiate from a contractile or quiescent phenotype to a proliferative or synthetic phenotype in response to various environmental stimuli [5]. PASMCs underwent a contractile-to-synthetic phenotypic switch, which was accompanied with an excess of proliferation and a primary cause underlying vascular remodeling [6]. Normally, phenotypic switch restricted the expression of contractile proteins, such as alpha smooth muscle actin (α-SMA), smooth muscle (SM) myosin heavy chain and SM-22α [7]. Currently, no available medical treatment is considered curative and lung transplantation continues to be an important therapeutic consideration in PH [1, 8]. Therefore, it will be significant to unveil the underlying molecular mechanisms, which will supply us with novel therapies for PH.

Long non-coding RNAs (lncRNAs) are non-protein-coding RNA segments with length of greater than 200 nucleotides [9]. In vascular system, accumulating evidence implied that lncRNAs could play a significant role in vascular pathophysiology [10]. Besides, many reports suggested relevance of lncRNAs in the vascular remodeling [11, 12]. However, the roles of lncRNAs to pulmonary vascular biology and to the pathogenesis of PH were yet to be investigated. The novel lncRNA gene Cancer Susceptibility Candidate 2 (CASC2) is located on chromosome 10 in humans and has been characterized as a tumor suppressor in human malignancies [13]. For example, it was reported that cell proliferation and metastasis were promoted by down-regulation of lncRNA CASC2 in both bladder and renal cell carcinoma [14, 15]. Given that PH and cancer were shared with some common features, like excessive cellular proliferation and apoptosis resistance, we were interested to unveil whether lncRNA CASC2 is involved in PH pathogenesis.

In this study, our results showed the dys-regulation of lncRNA CASC2 was involved in proliferation and phenotype switch of PASMCs to mediate the vascular remodeling of hypoxic-induced PH, which might provide novel strategies and insights for PH therapy.

## Methods and materials

### Cell culture and hypoxic induction

Human PASMCs were obtained from BeNa Culture Collection (Beijing, China), and the cells were cultured in 90% high glucose Dulbecco′s Modified Eagle′s Medium (DMEM) containing 10% fetal bovine serum (FBS) (Gibco, San Diego, NY, USA), 100 U/mL penicillin, and 100 mg/mL streptomycin in a humidified 5% CO_2_ at 37 °C. For the hypoxic induction, PASMCs were incubated in the hypoxic condition of 92% N_2_, 5% CO_2_ and 3% O_2_ for 48 h. Before hypoxic induction, PASMCs which went through 2 to 3 continuous passage were starved for 24 h in the DMEM medium without serum.

### Cell transfection

The recombinant plasmid pcDNA3.1-lncRNA CASC2 and pcDNA3.1 empty vector were provided by GenePharma (Shanghai, China). Before transfection, cells (1 × 10^5^) were cultured until cells reached 80% confluency. Cell transfection was conducted by using Lipofectamine™ 2000 transfection reagent (Invitrogen, CA, USA). Cells transfected with recombinant plasmids were cultured for 36 h to select cells in which lncRNA CASC2 was overexpressed.

### Real time-quantitative polymerase chain reaction (qRT-PCR)

Total RNA from PASMCs or rat pulmonary arterial tissues was extracted using the Trizol reagent and reversely transcribed into cDNA using Prime-Script RT-PCR kit (Takara, Dalian, China) following the manufacturer′s instructions. The primers were listed in Table 1. Thereafter, polymerase chain reaction was performed using a LightCycler 1.0 (Roche, Basel, Switzerland). The expression of both mRNAs and lncRNA was normalized against glyceraldehyde-3-phosphate dehydrogenase (GAPDH) and relatively quantified using ${2}^{-\Delta  \Delta  {C}_t}$ method.

**Table 1:** Primers for qRT-PCR

Gene		Sequence
CASC2	Forward	5′-TACAGGACAGTCAGTGGTGGTA-3′
	Reverse	5′-ACATCTAGCTTAGGAATGTGGC-3′
PCNA	Forward	5′-TTGCACGTATATGCCGAGACC-3′
	Reverse	5′-GGTGAACAGGCTCATTCATCTCT-3′
GAPDH	Forward	5′-GGTCACCAGGGCTGCTTTTA-3′
	Reverse	5′-GGATCTCGCTCCTGGAAGATG-3′

### Cell number assay

Cell number assay was performed using CyQuant cell proliferation assay kit (ThermoFisher Scientific, MA, USA) for assessment of cell proliferation. Cells (4 × 10^3^) with the corresponding treatments were incubated in a 96-well plate. After 48 h, freshly prepared 10 μL detection solution fluorescent-DNA-binding dye mixture (provided by kit) was added to each well. Afterwards, cells were incubated for another 5 min in dark. The optical density value was measured at 450 nm with a microplate reader (Beyotime, Shanghai, China).

### Western blot analysis

Proteins were extracted using nucleic/plasma protein extraction kit (Viagene, Tampa, FL, USA). Extracted proteins were separated by sodium dodecyl sulfate -polyacrylamide gel electrophoresis (SDS-PAGE) after quantification, and transferred onto a polyvinylidene fluoride membrane. Therefter, the membrane was rinsed with PBST buffer, blocked for 2 h in PBST containing 5% non-fat milk, and incubated with primary antibodies, including anti-α-SMA antibodies (1:1000; ab5694, Abcam, Cambridge, USA), anti-syndecan-1 antibodies (1:1000; ab181789, Abcam), anti-tropomysin antibodies (1:1000; ab133292, Abcam), anti-myocardin antibodies (1:1000; ab107301, Abcam), anti-PCNA antibodies (1:1000; ab29, Abcam), and anti-GAPDH antibodies (1:1000; ab8245, Abcam). Subsequently, the membrane was washed with PBST and then incubated with secondary antibodies goat anti-mouse IgG (1:1000; ab6785, Abcam). GAPDH served as internal control to normalize the expression levels of proteins. Blots were visualized using an ECL detection kit (Amersham Pharmacia Biotech, USA) and exposed to X-ray film.

### EdU (5-ethynyl-2′-deoxyuridine) staining

EdU staining was performed using Click iT™ EdU cell proliferation assay kit (Molecular Probes, Invitrogen). Cells were seeded into 96-well plates and stained with 50 μM EdU for 2 h. Afterwards, the cells were washed twice with PBS and fixed with 50 μL of fixation fluid (PBS + 4% polyoxymethylene) and incubated for 30 min. Finally, 100 μL of penetrant (PBS + 0.5% TritonX-100) was used to discolor cells 2 to 3 times (10 min per rinsing). Cell nuclei were stained with DAPI for 10 min. The results of cell staining were then examined using a florescence microscope (Olympus, Tokyo, Japan).

### Flow cytometry

Annexin V-FITC/propidium iodide (PI) apoptosis kit (BD Biosciences, CA, USA) was applied to evaluate cell apoptosis. Transfected PAMSCs were trypsinized, harvested, and then collected after centrifugation. Cells were stained with Annexin V and PI according to the instruction after washed by PBS (cold). The early as well as late apoptotic cells were detected using flow cytometry and Cell Quest Pro software (BD Bioscience).

### Wound healing assay

Wound healing assay was used to determine cell migration. Transfected PAMSCs were seeded in 6-well plates and cultured until cells reached 90–95% confluency. Cells were wounded using pipette tips and cultured after cell debris was washed by PBS. Wounded areas were photographed at 0 h and 24 h after wound and migratory distance of cells was analyzed.

### Establishment of rat PH models

Forty-eight male Wistar rats, aged nine-week-old and weighed initially 180–220 g, were used in the experiments. All animal experimental procedures were approved by the Animal Care and Ethics Committee of Chinese Academy of Medical Sciences and Peking Union Medical College. Rats were randomly divided into normoxia group (12 rats) with 21% O_2_ environment and hypoxia group (36 rats) with 12% O_2_ environment for 21 days, respectively. Thirty-six rats were then randomly divided into three groups, hypoxia group, hypoxia + p-CACS2 group, and hypoxia + pcDNA3.1 group, intravenously injected with cells treated with pcDNA3.1-CACS2 recombinant plasmid, pcDNA3.1 vector and saline of the same volume respectively every 72 h during 2 weeks. The injected rats were then kept in hypoxic environment. After 21 days of hypoxic induction, rats of four groups were anesthetized by urethane (1 g/kg) through an intraperitoneal injection for subsequent measurements, and sacrificed by cervical dislocation and rat pulmonary arterial tissues were isolated for the further studies.

### Hemodynamic measurements

After 21 days of hypoxic induction, rats of four groups were anesthetized and used for this assay. The heparinized PV-1 catheter was inserted into the pulmonary artery through the external jugular vein, right atrium, and right ventricle, and connected to a pressure transducer (Statham P23ID). And a multipurpose polygraph connected to the pressure transducer was used to measure the mean pulmonary arterial pressure (mPAP). Systemic blood pressure (SBP) was measured via carotid artery cannulation. The right ventricular (RV) wall was separated from the left ventricular (LV) wall and ventricular septum (IVS), and then severally weighed. Thereafter, the index of RV hypertrophy (RVI) was calculated by the weight ratio of right ventricle to left ventricle plus septum (RV/[LV + IVS]).

### Immunohistochemistry (IHC)

Paraffin-embedded specimens were sectioned at 4 μm thickness according to standard histopathological techniques. The endogenous peroxidase activity was blocked with 3% H_2_O_2_ for 10 min. The sections were stained for primary anti-α-SMA antibody (1:200; ab8207, Abcam) and incubated overnight at 4 °C. Thereafter, the secondary IgG antibody (1:1000; ab6785, Abcam) was added and then sections were incubated for another 30 min at 37 °C. Subsequently, sections were stained with diaminobenzidine (DAB) after incubation with secondary antibody. Non-specific binding was blocked by 5% normal goat serum for 10 min. IHC results were analyzed using NIH ImageJ v1.56 (National Institutes of Health, Bethesda, MD, USA).

### Pulmonary artery morphometry

Pulmonary artery morphometry was analyzed by Hematoxylin and eosin (HE) and Masson′s trichrome staining. For HE staining, pulmonary arterial tissues were fixed in 10% formaldehyde for 24 h, which was followed by dehydration, permeation, wax dip, paraffin embedding and then cut into 3 μm sections. Subsequently, the sections were stained with hematoxylin and eosin. The visual imaging system image-Pro plus 6.0 program was used to capture and analyze the image. The percentage of medial wall thickness to the external diameter (WT%) and the percentage of cross-sectional vessel wall area to the total area (WA %) of small pulmonary artery were calculated as morphometry parameters. For Masson trichrome staining, pulmonary arterial tissue samples were fixed in 4% paraformaldehyde, embedded in paraffin and sectioned. With standard histological techniques, samples were stained with Masson′s trichrome to detect collagen deposition in aorta sections. Light microscope images were captured with a color video camera (Olympus Microscope BX-51, Japan) and analyzed with image analysis software (Qianping Imaging, China).

### Statistical analysis

All statistical analyses were implemented using GraphPad Prism 6.0. Data was presented as mean ± standard deviation (mean ± SD), and one way analysis of variance (ANOVA) was used to evaluate statistical differences. *P* < 0.05 was considered significant throughout the study.

## Results

### Up-regulation of lncRNA CASC2 inhibited proliferation, migration, while enhanced apoptosis in hypoxia-induced PASMCs

**Fig. 1:**
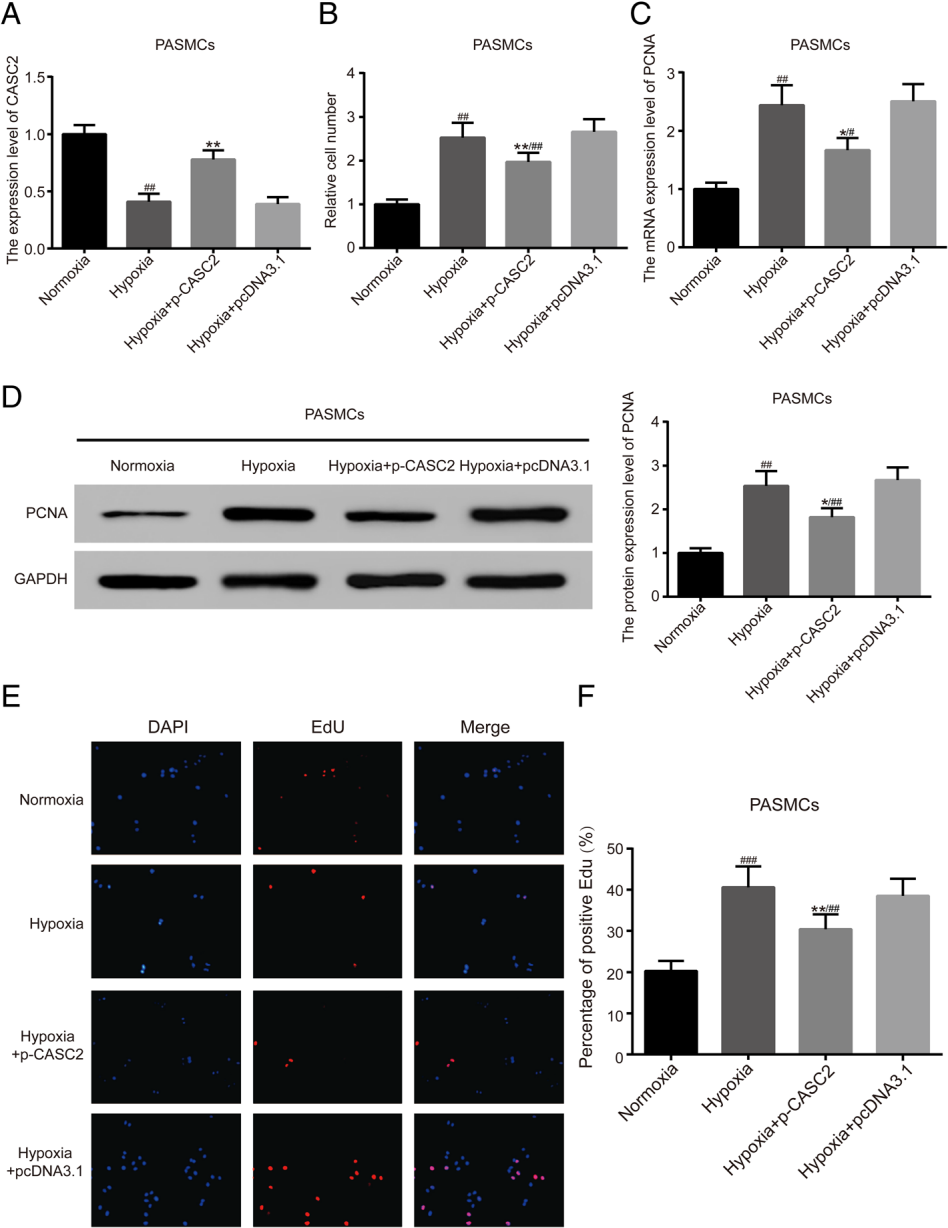
Up-regulation of lncRNA CASC2 inhibited proliferation in hypoxia-induced PASMCs. (**a**) The expression of lncRNA CASC2 was detected by qRT-PCR in PASMCs. (**b**) Cell number of PASMCs was counted by CyQuant cell proliferation assay kit. (**c**) The mRNA expression of PCNA was detected by qRT-PCR in PASMCs. (**d**) The protein expression of PCNA was detected by western blot analysis in PASMCs. (**e**, **f**) Cell proliferation status of PASMCs was assessed by EdU staining assay. ^###^*p* < 0.001, compared with normoxia group. ^##^ *p* < 0.01, compared with normoxia group. ^#^*p* < 0.05, compared with normoxia group. ^**^p < 0.01, ^*^p < 0.05, compared with hypoxia + pcDNA3.1 group

**Fig. 2:**
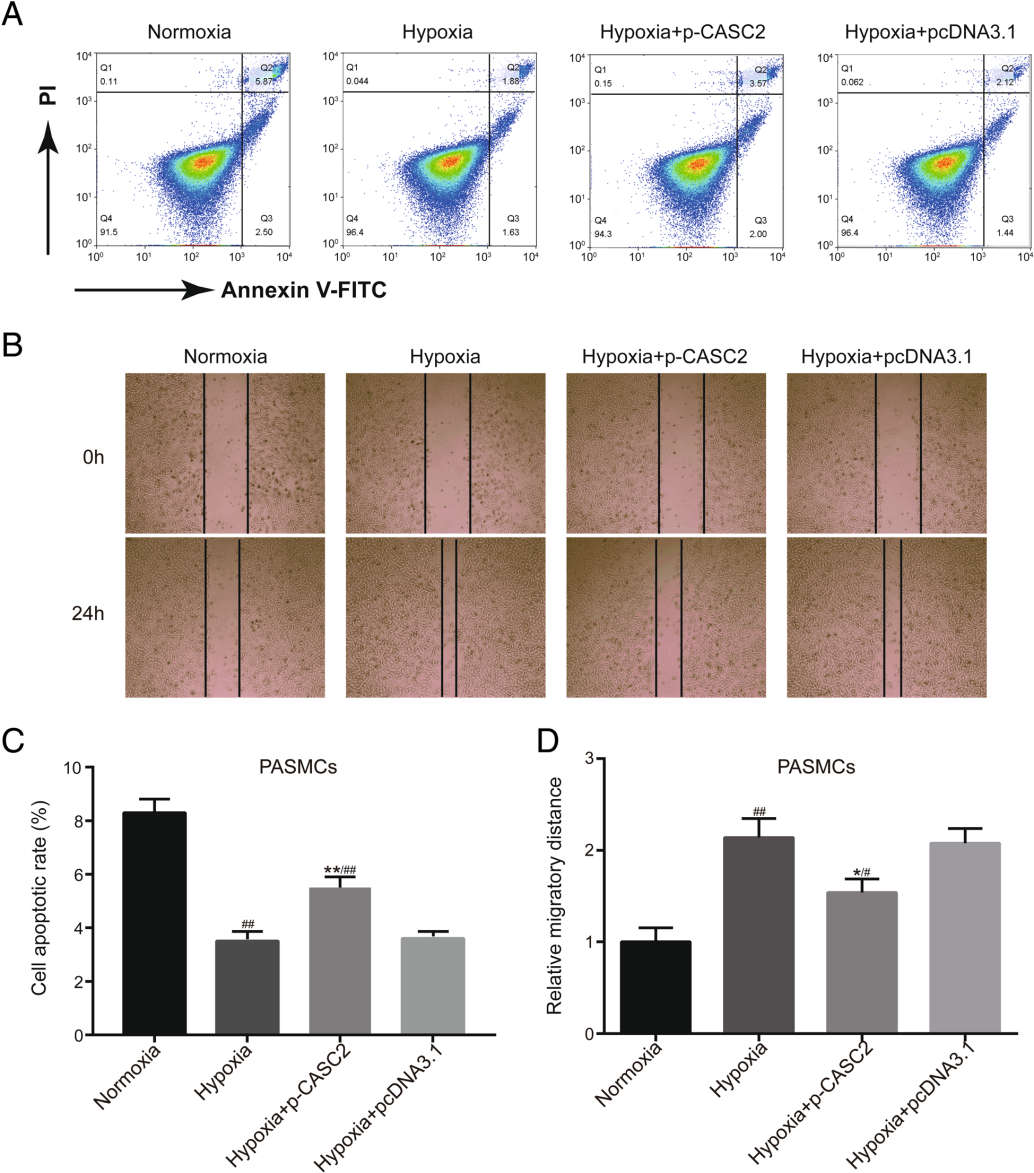
Up-regulation of lncRNA CASC2 enhanced apoptosis yet inhibited migration in hypoxia-induced PASMCs. (**a**, **c**) Cell apoptosis was detected using flow cytometry in PASMCs. (**b**, **d**) Cell migration was assessed by wound healing assay in PASMCs. ^##^*p* < 0.01, compared with normoxia group. ^#^*p* < 0.05, compared with normoxia group. **p < 0.01, ^*^p < 0.05, compared with hypoxia + pcDNA3.1 group

Cell transfection was performed to modulate the expression of lncRNA CASC2 in PASMCs under both normoxic condition and hypoxia condition, and qRT-PCR was employed for detection of transfection efficiency in PASMCs. The results of qRT-PCR verified that the expression of lncRNA CASC2 was greatly up-regulated in normoxia + p-CASC2 group compared with normoxia group. Besides, relative expression of lncRNA CASC2 was almost not affected in normoxia + pcDNA3.1 group compared with normoxia group (Additional file 1: Figure S1). Above all, our transfection was successful and p-CASC2 could be utilized for overexpression of lncRNA CASC2 in this study. Besides, relative expression of lncRNA CASC2 was significantly decreased in hypoxia group compared with normoxia group. Besides, lncRNA CASC2 was up-regulated in hypoxia + p-CASC2 group compared with hypoxia+ pcDNA3.1 group (Fig. 1a). In addition, the expression of lncRNA CASC2 was slightly down-regulated in hypoxia + p-CASC2 group compared with normoxia group. To assess cell proliferation ability of PASMCs, cell number assay was performed and the expression of cell proliferation marker PCNA was detected using both qRT-PCR and western blot. The results of cell number assay verified that cell proliferation ability was enhanced in hypoxia group compared with normoxia group. Besides, cell proliferation ability was suppressed in hypoxia + p-CASC2 group compared with hypoxia+ pcDNA3.1 group. In addition, enhancement of cell proliferation ability induced by hypoxia was partly alleviated by overexpression of lncRNA CASC2 in hypoxia + p-CASC2 compared with normoxia group (Fig. 1b). In addition, PCNA was usually employed for reflecting cell proliferation ability. MRNA expression of PCNA was increased in hypoxia group compared with normoxia group. Besides, overexpression of lncRNA CASC2 greatly down-regulated mRNA expression of PCNA in hypoxia + p-CASC2 group compared with hypoxia + pcDNA3.1 group. In addition, up-regulation of mRNA expression of PCNA caused by hypoxia was partly attenuated by overexpression of lncRNA CASC2 in hypoxia + p-CASC2 group compared with normoxia group (Fig. 1c). The results of western blot analysis were in line with the results as indicated by qRT-PCR (Fig. 1d). Thereafter, EdU staining was employed for detection of cell proliferation ability likewise. Percentage of positive EdU was increased in hypoxia group compared with normoxia group. However, percentage of positive EdU in hypoxia + p-CASC2 group was lower than that in hypoxia + pcDNA3.1 group (Fig. 1e, f). In addition, flow cytometry indicated that cell apoptosis was suppressed by hypoxia, and overexpression of lncRNA CASC2 enhanced cell apoptosis in hypoxia + p-CASC2 group compared with hypoxia + pcDNA3.1 group (Fig. 2a, c). In addition, down-regulation of cell apoptotic rate by hypoxia was partially relieved by up-regulation of lncRNA CASC2 in hypoxia + p-CASC2 group compared with normoxia group. The results of migration assay indicated that cell migration was promoted by hypoxia and the promotion was partly relieved by the up-regulation of lncRNA CASC2 in hypoxia-induced PASMCs (Fig. 2b, d). Taken together, lncRNA CASC2 was down-regulated in hypoxia-induced PASMCs compared with PASMCs under normoxia condition, and up-regulation of lncRNA CASC2 inhibited proliferation, migration, while enhanced apoptosis in hypoxia-induced PASMCs.

### LncRNA CASC2 suppressed phenotype switch induced by hypoxia in PASMCs

**Fig. 3:**
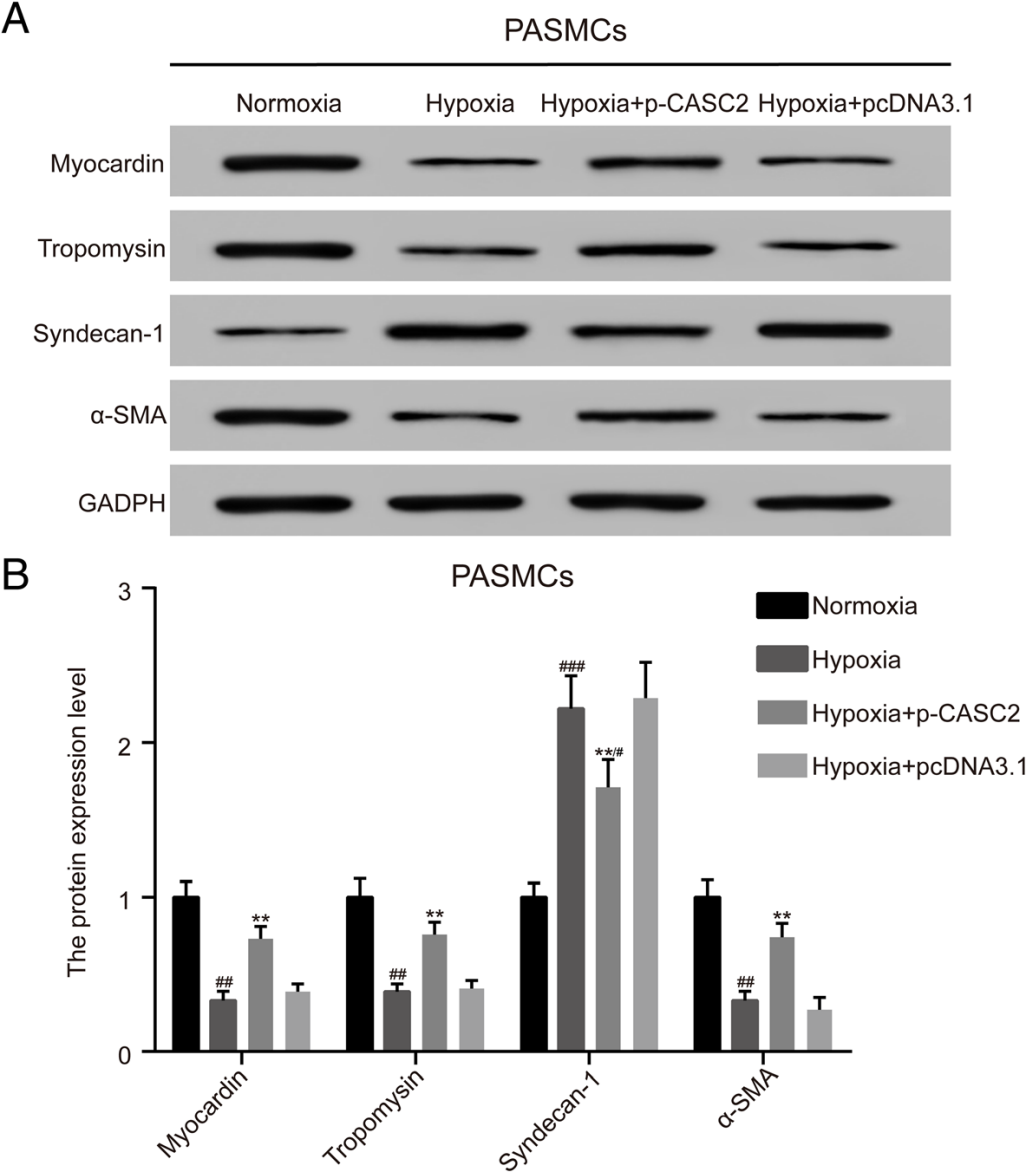
LncRNA CASC2 suppressed phenotype switch induced by hypoxia in PASMCs. (**a**-**b**) The expression of phenotype switch marker proteins including myocardin, tropomysin, syndecan-1 and α-SMA was detected by western blot analysis in PAMSCs. ^##^*p* < 0.01. ^#^*p* < 0.05, compared with normoxia group. ^###^*p* < 0.001, compared with normoxia group. **p < 0.01, compared with hypoxia + pcDNA3.1 group

The expression of phenotype switch markers, including myocardin, tropomysin and α-SMA was detected by western blot analysis in PASMCs, and the results showed that the expression of myocardin, tropomysin and α-SMA was decreased, while the syndecan-1 was increased in hypoxia group compared with normoxia group. Besides, the expression of myocardin, tropomysin and α-SMA were higher, while the expression of syndecan-1 was lower in hypoxia + p-CASC2 group than that in hypoxia + pcDNA3.1 group (Fig. 3a, b). Besides, down-regulation of myocardin, tropomysin and α-SMA, as well as up-regulation of syndecan-1 caused by hypoxia was be partially retrieved by overexpression of lncRNA CASC2 in hypoxia + p-CASC2 group compared with normoxia group. In brief, up-regulation of lncRNA CASC2 suppressed the phenotype switch induced by hypoxia in PASMCs.

### LncRNA CASC2 suppressed progression of right ventricular hypertrophy caused by hypoxia in rat pulmonary arterial tissues

**Fig. 4:**
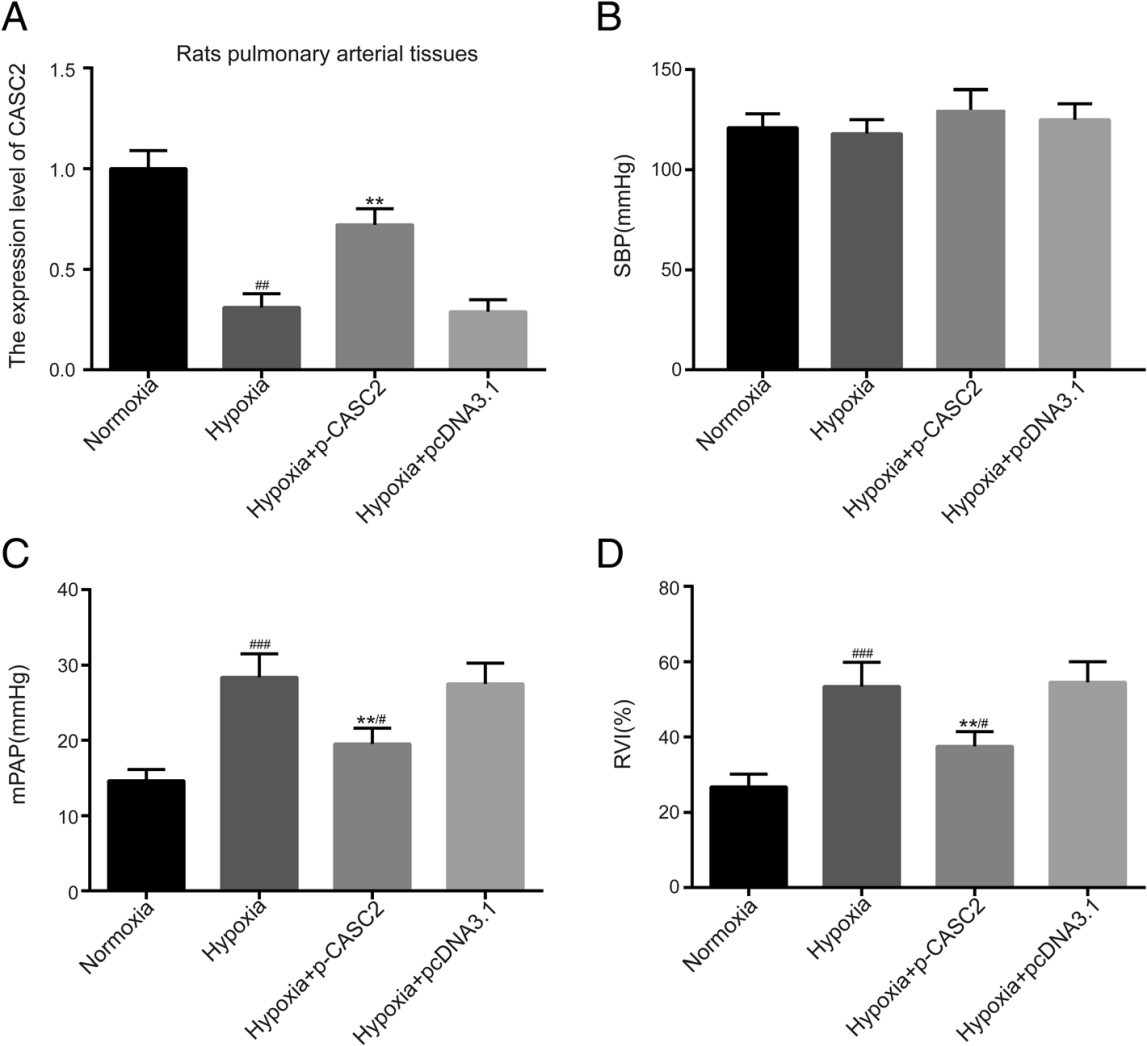
LncRNA CASC2 was down-regulated in hypoxia-induced rats and reversed the increase of mPAP and progression of right ventricular hypertrophy caused by hypoxia. (**a**) The expression of lncRNA CASC2 was detected by qRT-PCR in rat pulmonary arterial tissues. (**b**) Systemic blood pressure (SBP) was assessed in normoxia and hypoxia-induced rats. (**c**) Mean pulmonary arterial pressure (mPAP) was detected in normoxia and hypoxia-induced rats. (**d**) The right ventricular hypertrophy index (RVI) was calculated in normoxia and hypoxia-induced rats. ^#^*p* < 0.05, ^##^*p* < 0.01, ^###^*p* < 0.001, compared with normoxia group. **p < 0.01, compared with hypoxia + pcDNA3.1 group

SBP, mPAP and RVI were quantified for validation of progression of right ventricular hypertrophy caused by hypoxia in rat pulmonary arterial tissues in this study. The expression of lncRNA CASC2 was significantly decreased in hypoxia group compared with normoxia group, and lncRNA CASC2 was significantly increased in Hypoxia + p-CASC2 group compared with Hypoxia + p-CDNA3.1 group (Fig. 4a). Besides, overexpression of lncRNA CASC2 partly restored the down-regulation of lncRNA CASC2 caused by Hypoxia in Hypoxia + p-CASC2 group compared with normoxia group. Besides, Hypoxia or up-regulation of lncRNA CASC2 almost had no effect on SBP (Fig. 4b). In addition, mPAP and RVI were significantly increased in hypoxia group compared with the normoxia group (Fig. 4c, d), which indicated that the hypoxia-induced PH rat model was successfully established. Furthermore, hypoxia-induced PH rats were intravenously injected into PASMCs with or without lncRNA CASC2 overexpression for modulation of the expression of lncRNA CASC2. In addition, mPAP and RVI were decreased in hypoxia + p-CASC2 group compared with the hypoxia + pcDNA3.1 group. Besides, up-regulation of mPAP and RVI caused by Hypoxia was partly attenuated by overexpression of lncRNA CASC2 in Hypoxia + p-CASC2 group compared with normoxia group, indicating suppressive role of lncRNA CASC2 in progression of right ventricular hypertrophy. In short, lncRNA CASC2 suppressed progression of right ventricular hypertrophy caused by hypoxia in rat pulmonary arterial tissues.

### LncRNA CASC2 inhibited cell proliferation in hypoxia-induced rat pulmonary arterial tissues

**Fig. 5:**
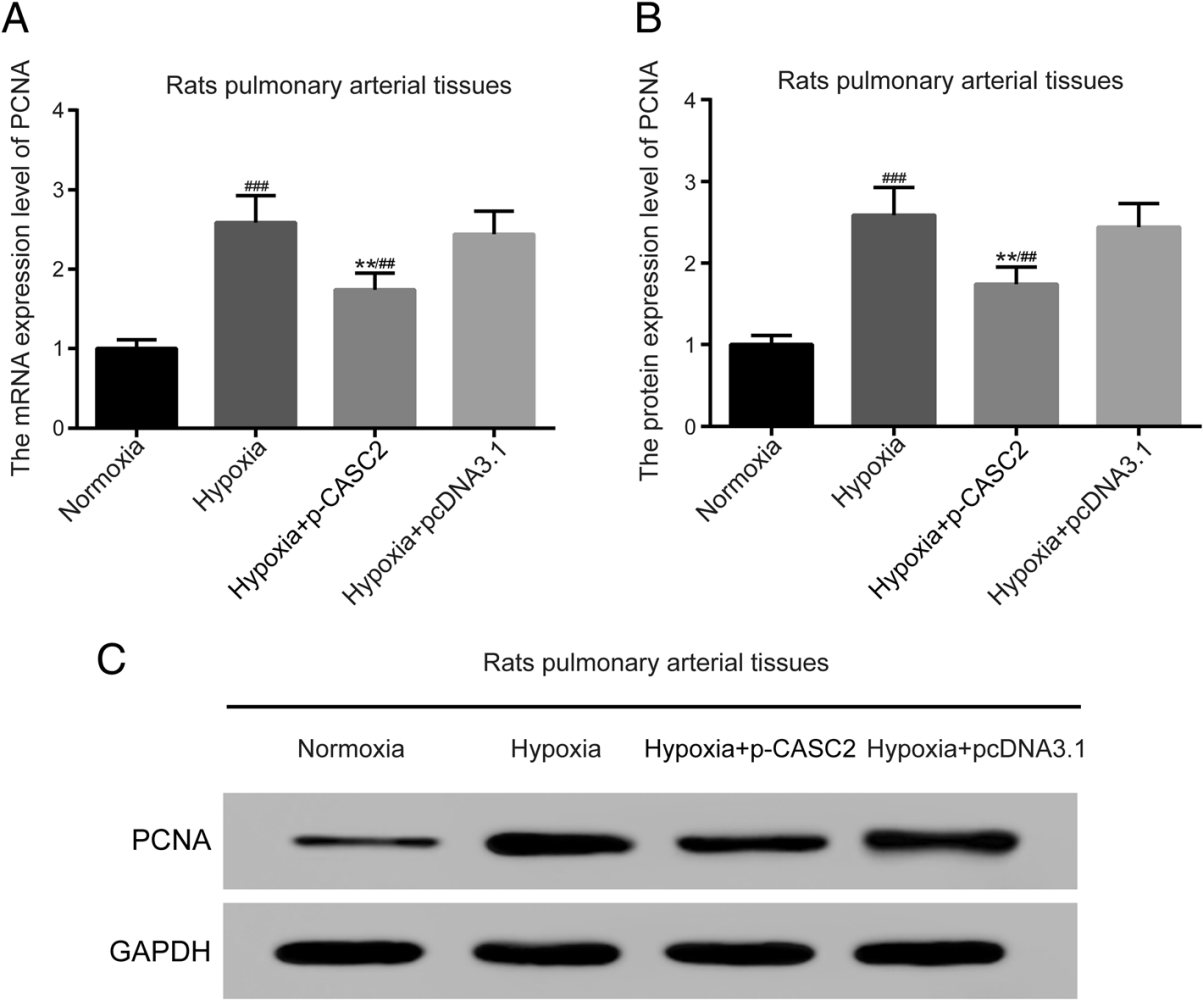
LncRNA CASC2 inhibited hypoxia-induced cell proliferation in rat pulmonary arterial tissues. (**a**) The mRNA expression of PCNA was detected by qRT-PCR in rats′ PAs tissues. (**b**, **c**) The protein expression of PCNA was detected by western blot analysis in rat pulmonary arterial tissues. ^##^*p* < 0.01, compared with normoxia group. ^###^*p* < 0.001, compared with normoxia group. **p < 0.01, compared with hypoxia + pcDNA3.1 group

QRT-PCR and western blot analysis were employed for detecting relative mRNA and protein expression of cell proliferation marker PCNA to assess the cell proliferation status in rat pulmonary arterial tissues. The results of qRT-PCR indicated that PCNA was up-regulated in hypoxia group compared with normoxia group. Besides, mRNA expression of PCNA was down-regulated in hypoxia + p-CASC2 group compared with hypoxia + p-cDNA3.1 group (Fig. 5a). In addition, up-regulation of lncRNA CASC2 partly alleviated the up-regulation of PCNA induced by hypoxia in hypoxia + p-CASC2 group compared with normoxia group. Besides, the results of western blot analysis were in line with the results indicated by qRT-PCR (Fig. 5b, c). To sum up, lncRNA CASC2 inhibited cell proliferation in hypoxia-induced rat pulmonary arterial tissues.

### LncRNA CASC2 suppressed phenotype switch and vascular remodeling in hypoxia-induced rat pulmonary arterial tissues

**Fig. 6:**
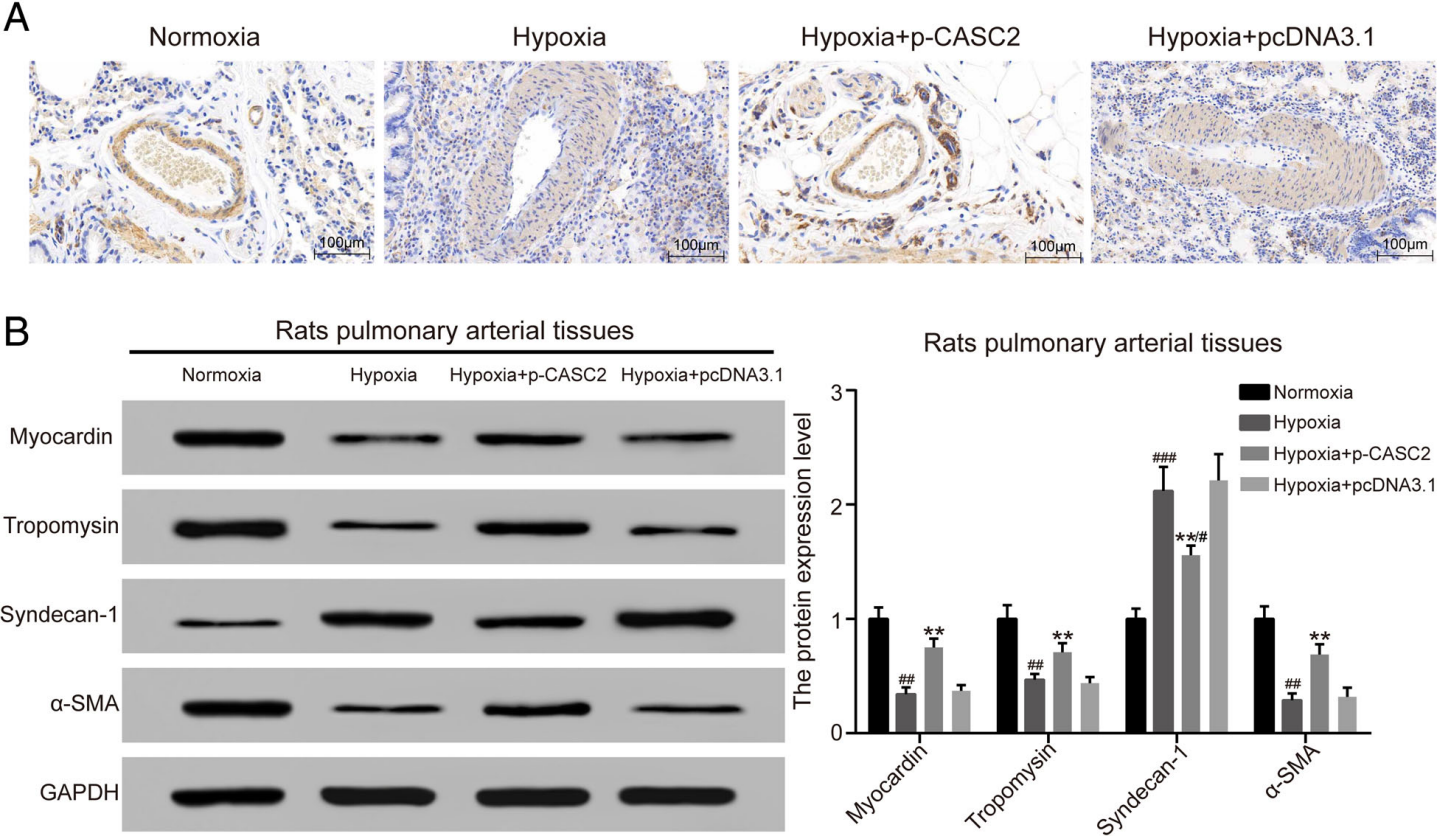
LncRNA CASC2 suppressed phenotype switch of hypoxia-induced rat pulmonary arterial tissues. (**a**) Immunohistochemical detection of phenotype switch marker protein α-SMA was performed in rat pulmonary arterial tissues. (**b**) The expression of phenotype switch markers including myocardin, tropomysin, syndecan-1 and α-SMA was detected by western blot analysis in rat pulmonary arterial tissues. ^#^*p* < 0.05, ^##^*p* < 0.01, ^###^*p* < 0.001, compared with normoxia group. **p < 0.01, compared with hypoxia + pcDNA3.1 group

**Fig. 7:**
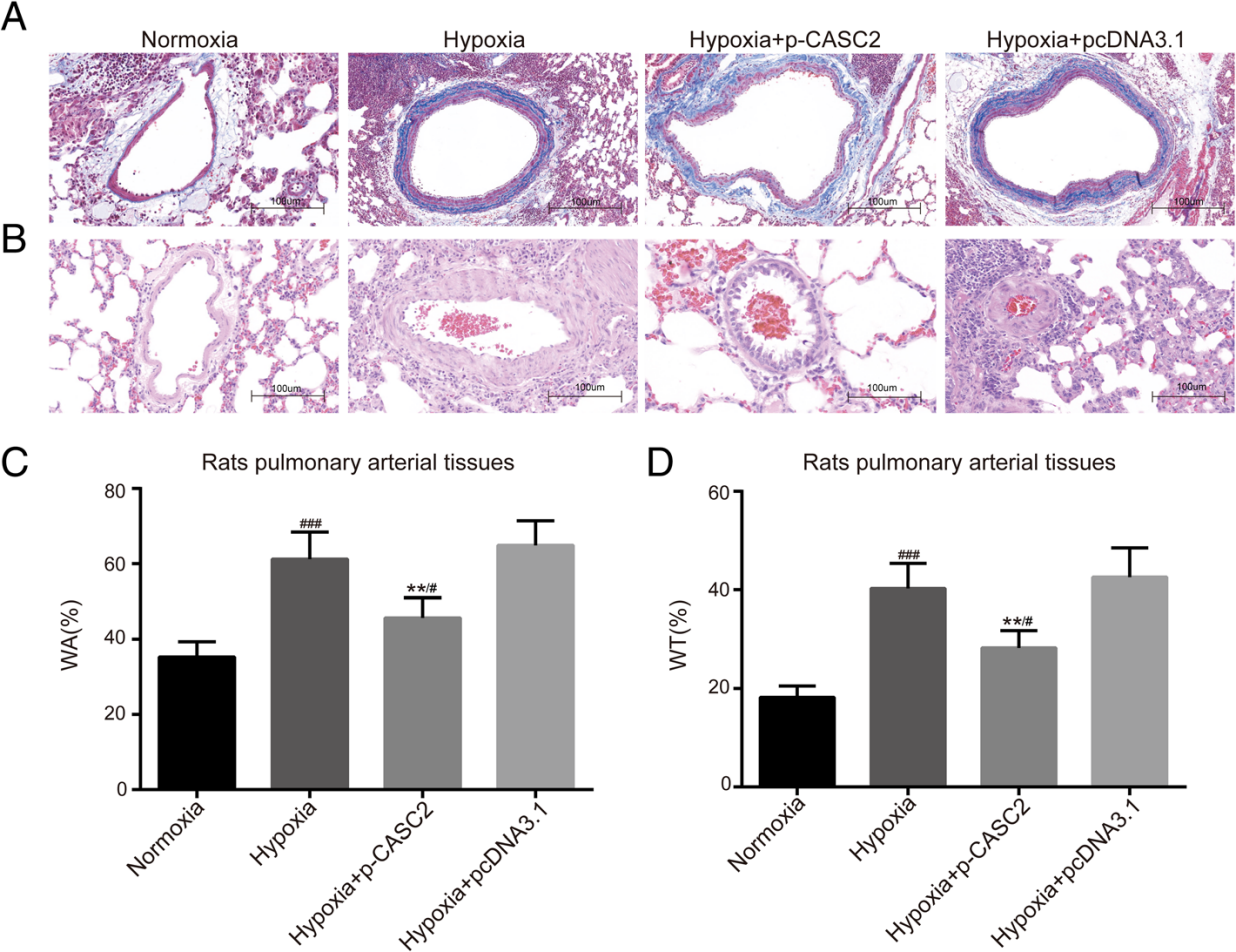
LncRNA CASC2 suppressed vascular remodeling of hypoxia-induced rat pulmonary arterial tissues. (**a**) Tissue fibrosis was measured by Masson′s trichrome staining in rat pulmonary arterial tissues and the collagen fiber was stained with blue. (**b**) The morphometric analysis of rat pulmonary arterial tissues was performed by hematoxylin and eosin (HE) staining. The parameters of pulmonary vascular cross-section were calculated by measuring the medial wall thickness, total vessel wall thickness, cross-sectional vessel area and wall area. (**c**) The percentage of medial wall thickness to the external diameter (WT%) was calculated in rats′ PAs tissues. (**d**) The percentage of cross-sectional total vessel wall area to the total area (WA %) was calculated in rat pulmonary arterial tissues. ### *p* < 0.001, compared with normoxia group. ^**^ *p* < 0.01, compared with hypoxia + pcDNA3.1 group

The expression of phenotype switch marker s was detected by IHC and western blot analysis in hypoxia-induced rat pulmonary arterial tissues. IHC results showed that α-SMA was decreased in hypoxia group compared with normoxia group. Besides, the expression α-SMA was increased in hypoxia + p-CASC2 group compared with hypoxia + pcDNA3.1 group (Fig. 6a). Down-regulation of α-SMA induced by hypoxia was partially relieved by up-regulation of lncRNA CASC2 in hypoxia + p-CASC2 group compared with normoxia group. Besides, the results of western blot analysis showed that the expression of myocardin, tropomysin and α-SMA were decreased, while expression of syndecan-1 was increased in hypoxia group compared with normoxia group. Besides, the expression of myocardin, tropomysin and α-SMA were increased, while the expression of syndecan-1 was decreased in hypoxia + p-CASC2 group compared with hypoxia + pcDNA3.1 group (Fig. 6b). Down-regulation of myocardin, tropomysin and α-SMA, as well as up-regulation of syndecan-1 induced by hypoxia was partly attenuated by up-regulation of lncRNA CASC2 in hypoxia + p-CASC2 group compared with normoxia group. Furthermore, Pulmonary artery morphometry was assessed by Masson′s trichrome staining and HE staining. The results of Masson′s trichrome staining showed that total of collagen fiber in aorta was greatly increased in hypoxia group compared with normoxia group. Besides, total of collagen fiber in aorta was decreased in hypoxia + p-CASC2 group compared with hypoxia + pcDNA3.1 group. Furthermore, up-regulation of total of collagen fiber in aorta by hypoxia was partially attenuated by overexpression of lncRNA CASC2 in hypoxia + p-CASC2 group compared with normoxia group (Fig. 7a). As shown in Fig. 7b, the results of HE staining indicated the pathological progress in hypoxia group compared with normoxia group. Besides, WA and WT and fibrosis of pulmonary arteries were significantly increased in hypoxia group compared with normoxia group. Meanwhile, compared with hypoxia + pcDNA3.1 group, WAWT and fibrosis of pulmonary arteries were notably decreased in hypoxia + p-CASC2 group (Fig. 7c, d). Besides, up-regulation of WA and WT induced by hypoxia was partly relieved by up-regulation of lncRNA CASC2 in hypoxia + p-CASC2 group compared with normoxia group. Above all, these results indicated that lncRNA CASC2 suppressed the phenotype switch and the vascular remodeling of hypoxia-induced rat pulmonary arterial tissues.

## Discussion

Increased pulmonary arterial pressure and vascular remolding were the characteristics of PH, which finally leads to deadly right heart failure [16]. The remodeling of the pulmonary artery mainly involves proliferation, and migration of PASMCs induced by hypoxic exposure [17]. Despite the availability of multiple agents for the treatment of PH, PH remains a progressive disease with unacceptably high morbidity and mortality [18].

The phenotypic switch of PASMCs is the conversion from contractile/differentiated phenotype to synthetic/dedifferentiated phenotype, which leads to vascular remodeling and contributes to PH development [19]. In addition, the phenotypic switch of PASMCs from a contractile to proliferative phenotype is inevitable for any physiological or pathological vascular remodeling process to occur [20]. It has been demonstrated that pulmonary vascular remodeling is associated with the abnormal proliferation of PASMCs [20]. Considering PH is a disease with quasi-malignant growth of cells in the pulmonary vascular wall, therapies are being developed which inhibit hypertrophy and angiogenesis, and promote apoptosis, but the inherent danger of these therapies is a further compromise to the already ischemic, fibrotic and dysfunctional right ventricle [21]. Previous studies have investigated the mechanism of vascular remolding in PH. For example, Liu et al. showed that lncRNA TCONS_00034812 modulated PASMCs proliferation and apoptosis and participated in vascular remodeling during the course of PH [16]. Fernandez et al. reported that knockout of SMAD3 enhanced vascular remodeling in PH [22]. However, their studies on vascular remodeling paid attention to cell proliferation and morphological detection, rarely investigated the phenotypic switch of PASMCs. Apart from the findings that hypoxia enhanced cell proliferation, migration and decreased cell apoptosis consistent with other studies, our results also showed a molecular mechanism that lncRNA CASC2 was involved in vascular remolding of PH, precisely, phenotypic switch of PH.

Recently, it has been reported that lncRNA CASC2 was involved in regulating the carcinogenesis and suppressing tumor progression [23]. For instance, down-regulation of lncRNA CASC2 promoted tumorigenesis in thyroid carcinoma [24], and overexpression of lncRNA CASC2 inhibited cell proliferation and angiogenesis in gastric cancer [25]. It is recognized that the vascular remodeling of hypoxia-induced PH is tightly linked to aberrant PASMCs proliferation, which is considered as a tumor-like biologic behavior. However, the expression and function of lncRNA CASC2 in PH remain unclear. It has been demonstrated that the expression of lncRNA CASC2 was down-regulated and lncRNA CASC2 inhibited the cancer cells proliferation, invasion, migration and viability in osteosarcoma, gastric cancer and esophageal carcinoma [26–28]. Thus, we hypothesized that lncRNA CASC2 exerted its function in hypoxia-induced PASMCs proliferation, apoptosis and migration. In our study, we also found a significant down-regulation of lncRNA CASC2 in PAs tissues of the hypoxia-induced rats and in the hypoxia-induced PASMCs. Moreover, we demonstrated that lncRNA CASC2 suppressed hypoxia-induced PASMCs proliferation, migration and enhanced hypoxia-induced PASMCs apoptosis. In addition, it has been reported that lncRNA CASC2 inhibited metastasis and epithelial to mesenchymal transition of hepatocellular carcinoma and lung adenocarcinoma cells [23, 29]. Nevertheless, our results from both in vivo and in vitro studies demonstrated that lncRNA CACS2 inhibited the phenotypic switch of hypoxia-induced PASMCs. Significantly, our study for the first time provide direct evidences at the molecular, cellular, organic, and animal levels to indicate that the functional involvement of lncRNA CASC2 in PH. Certainly, the underlying mechanisms of lncRNA CASC2 inhibiting hypoxia-induced PASMC proliferation, migration and phenotypic switch are expected to be investigated in depth.

## Conclusions

We demonstrated that up-regulating the expression of lncRNA CASC2 suppressed proliferation, migration and phenotypic switch, and promoted apoptosis in hypoxia-induced PASMCs and rats PAs tissues. In short, lncRNA CASC2 was involved in the vascular remolding of PH, and lncRNA CASC2 might serve as a new therapeutic target for PH.

## Additional file

Additional file 1: **Figure S1.** LncRNA CASC2 were successfully up-regulated by recombinant plasmid in PASMCs under normoxic condition. The expression of lncRNA CASC2 was detected by qRT-PCR in PASMCs under normoxic condition. ^##^*p* < 0.01, compared with normoxia group. **p < 0.01, compared with hypoxia + p-CASC2 group. (TIF 140402 kb)

## Abbreviations

ANOVA: Analysis of variance
cDNA: Complementary DNA
DAB: Diaminobenzidine
DMEM: Dulbecco′s Modified Eagle′s Medium
ECL: Electro-Chemi-Luminescence
EdU: 5-ethynyl-2′-deoxyuridine
FBS: Fetal bovine serum
GAPDH: Glyceraldehyde-3-phosphate dehydrogenase
HE: Hematoxylin and eosin
IgG: Immunoglobulin G
IVS: Ventricular septum
lncRNA: Long non-coding RNA
LV: Left ventricular
mPAP: Mean pulmonary arterial pressure
PAH: Pulmonary Arterial Hypertension
PAs: Pulmonary arteries
PASMCs: Pulmonary Artery Smooth Muscle Cells
PCNA: Proliferating cell nuclear antigen
PH: Pulmonary hypertension
PI: Propidium iodide
qRT-PCR: Real time-quantitative polymerase chain reaction
RV: Right ventricular
RVI: the index of RV hypertrophy
SBP: Systemic blood pressure
SDS-PAGE: Sodium dodecyl sulfate polyacrylamide gel electrophoresis
SM: Smooth muscle
α-SMA: Alpha smooth muscle actin
